# Supplementary material for: Mechanically induced correlated errors on superconducting qubits with relaxation times exceeding 0.4 ms
Source: Nat Commun. 2024 May 10;15:3950. doi: 10.1038/s41467-024-48230-3 (PMC11087564; doi:10.1038/s41467-024-48230-3)
Supplement: Supplementary file 1 — Supplementary information [file 41467_2024_48230_MOESM1_ESM.pdf]

Supplementary information for:
Mechanically induced correlated errors on superconducting qubits with relaxation times exceeding 0.4 milliseconds

Shingo Kono†,[1,2,*] Jiahe Pan,[1,2,*] Mahdi Chegnizadeh,[1,2,*] Xuxin Wang,[1,2] Amir Youssefi,[1,2] Marco Scigliuzzo,[1,2] and Tobias J. Kippenberg‡[1,2]

[1] *Institute of Physics, Swiss Federal Institute of Technology Lausanne (EPFL), Lausanne, Switzerland*
[2] *Center for Quantum Science and Engineering, EPFL, Lausanne, Switzerland*

SUPPLEMENTARY NOTE 1. FABRICATION DETAILS

A. Fabrication process flow

Wafer information: We use (100)-oriented intrinsic float zone double-side polished silicon wafers with a high resistivity (> 10 kΩ-cm), supplied from Siegert. The thickness of the wafers is 525 μm, while the diameter is 4 inches.

Substrate preparation: Prior to the Nb deposition, the wafers are first cleaned by Piranha solution, which is dedicated to removing the organic contamination on the substrate. This is done by dipping the wafer into two identical and successive baths of sulfuric acid (H_2SO_4 96 % at 100°C) activated by hydrogen peroxide (H_2O_2 30 %) for 5 minutes each, followed by dipping in two DI water baths for 4 minutes each and spin-drying. Afterward, the wafers are dipped in buffered HF solution (NH_4F 40 % and HF 50 % with a volume dilution ratio of 7:1) for 5 minutes to remove the silicon oxide on the surface, followed by DI water washing and spin-drying. The cleaned wafers are then quickly transferred to the load-lock chamber of the sputtering tool (in less than 3 minutes) to avoid re-oxidation.

Deposition of niobium: Immediately after native oxide removal, we deposit 150-nm Nb thin film using DC sputtering (Pfeiffer SPIDER Sputtering system). The argon flow rate is optimized such that the thin film has around -100 MPa stress [1], which is measured by comparing the wafer bow before and after the sputtering. The film stress is -130 MPa stress for the multi-qubit device used in the main experiment.

Niobium patterning and etching: To pattern large structures including transmon pads, resonators, and coplanar waveguides, the wafer undergoes a dehydration bake, followed by coating with 1.5-μm AZ ECI 3007 photo-resist and baking at 100°C for 2 minutes (automatic coater, ACS200 Gen3). The resist is patterned using a direct laser writer (MLA 150) and developed with AZ 726 MIF (an organic solution based on TMAH) after post-exposure baking at 110°C for 1 minute (ACS200 Gen3). Prior to the Nb etching, we perform 10-s oxygen plasma (Tepla GiGAbatch) at 200 W power with a gas flow and a gas pressure of 200 sccm and 0.5 mbar, respectively, to remove the resist residues.

The Nb film is dry-etched with inductively-coupled SF_6 plasma while the substrate is cooled with helium from the back side (SPTS APS). The pressure of the chamber is 2 mTorr, the gas flow is 40 SCCM, and the RF source power is 250 W. The etching time is determined every time by monitoring the intensity of the 440-nm spectral line which corresponds to SiF (the by-product of Si etching), normally resulting in about 70 seconds. The etching is manually interrupted 5 seconds after a sharp increase in the intensity, i.e., the signature of the end of the Nb etching. The additional etching time of 5 seconds ensures that the Nb films are fully etched, leading to an approximately 150-nm over-etching of the silicon substrate (see Fig. S2b). Before starting the Nb etching for the actual wafer, we clean the chamber with oxygen plasma for 5 minutes. Then, we run an identical Nb etching process with a dummy silicon wafer for 2 minutes.

The photo-resist is removed with the following procedures. First, the surface of the resist denatured by the Nb etching is removed by a low-power oxygen plasma (200 W) for 2 minutes (Tepla GiGAbatch). The time is well calibrated so that not all the resist is removed with the oxygen plasma. The wafer is then dipped in a clean 1165 remover inside a 60°C water bath for 5 minutes with high-power sonication to remove most of the resist. Successively, the wafer is dipped in another clean 1165 remover solution and kept overnight at room temperature. The beaker containing the wafer and remover is put inside a 60°C water bath, followed by a strong sonication for 5 minutes. The wafer is cleaned in acetone and IPA inside the water bath with high-power sonication for 3 minutes each, followed by drying with a nitrogen gun.

Electron-beam lithography: Prior to the e-beam resist coating, we use low-power oxygen plasma (200 W) for 1 minute to remove any possible resist residues. The wafer is then dipped into HF acid (1% diluted) for 5 minutes to remove the oxide layers on the Si and Nb surfaces, followed by dipping in two DI water baths and spin-drying.

Afterward, we immediately coat the wafer with bilayer e-beam resist using the following procedures. First, a 500-nm MMA EL9 resist layer is coated, followed by bak-

* These authors contributed equally.
Corresponding authors:
† shingo.kono@epfl.ch
‡ tobias.kippenberg@epfl.ch

ing at 180°C for 5 minutes. Then, a 500-nm PMMA 495 A8 resist layer is coated, followed by baking at 180°C for 5 minutes.

The e-beam lithography is done with a beam diameter of approximately 4 nm, enabled with a 100-kV acceleration voltage and a 200-pA current (Raith EBPG5000+). During the exposure, two doses are used for making the resist structures for the Manhattan process with in-situ bandage pads [2]. First, a high dose (1600 $\mu C/cm^2$) is used to expose PMMA for defining the junction structure, while the proximity effect also exposes MMA, resulting in undercuts around the exposed area. Second, a low dose (350 $\mu C/cm^2$) is used to expose MMA at the tips of all the line structures for making undercuts to refrain Al films from being deposited on the MMA side walls. After the exposure, the resist is developed using a room-temperature solution of MIBK:IPA (1:3) for 2 minutes, followed by dipping in IPA for 1 minute and drying with a nitrogen gun.

Shadow evaporation: We use Plassys MEB550SL3, which is an ultra-high vacuum 3-chamber system dedicated to shadow evaporation for fabricating Josephson junctions. This system has a separate load-lock chamber equipped with argon ion milling and UV-lamp to generate ozone for ashing, while it has an evaporation chamber and an oxidation chamber, separately. It can transfer a wafer between the three chambers without breaking the vacuum.

The wafer with the bilayer resist coating is loaded in the load-lock chamber and is pumped it for 10 hours (pressure $< 10^{-7}$ Torr). The recipe begins with generating ozone for 1 minute to remove the resist residues on the Si surface. To ensure no resist residues, the ashing time is calibrated such that e-beam resists are etched by approximately 5 nm.

The wafer is then transferred to the evaporation chamber for fabricating Josephson junctions. Prior to every Al evaporation, we evaporate titanium at a rate of 0.2 nm/min for 2 minutes (with the closed shutter) and wait for 4 minutes such that the chamber pressure becomes below 2×10^{-9} Torr. In our e-beam lithographic design, we can deposit aluminum lines selectively by changing the in-plane rotation angle according to the angle of the line patterns to be used. To deposit the bottom layer of the junction, the wafer is tilted by $\theta = 45°$ without an in-plane rotation ($\phi = 0°$), and Al is evaporated by 40 nm at a deposition rate of 0.5 nm/sec.

The wafer is then transferred to the oxidation chamber to make the oxide layer. The oxidation time and pressure are calibrated as 10 minutes and 0.07 Torr, respectively, to obtain the target junction resistance of ≈ 7 kΩ.

Afterward, the wafer is returned to the evaporation chamber and tilted by $\theta = 45°$, where Al is evaporated by 30 nm two times at $\phi = \pm 90°$, respectively. The two evaporations are useful to cover all the faces of the oxidized bottom layer to stabilize the junction resistance.

Before connecting the junction to the Nb pads, we have to remove the niobium oxide from the connection parts by using argon ion milling in the load-lock chamber. To this end, the wafer is transferred to the load-lock chamber and tilted by 45°, where argon milling is performed for 4 minutes each at two in-plane rotation angles of $\phi = -45°$ and 135°, respectively. The time and power of the milling are calibrated to assure removing all the niobium oxide layers and connecting the Al leads to the Nb pads without a resistance layer in between.

Next, the wafer is transferred to the evaporation chamber and tilted by 45°, where 30/100/100-nm Al evaporations are performed at $\phi = 135°/-45°/135°$, respectively. The first 30-nm Al evaporation is required to avoid the disconnectivity in Al leads due to the shadowing effect, while two 100-nm Al evaporation is used for connecting the Al leads to the Nb pads above the over-etched Si substrate.

The final step is to transfer the wafer to the load-lock chamber and oxidize it with high-purity oxygen at 15 Torr for 10 minutes for making a clean oxide layer as a protection barrier before taking out the wafer from the chamber.

Lift-off: After the evaporation, the wafer is dipped and kept in remover 1165 for 6 hours at room temperature. High power sonication is then performed inside a 60°C water bath for 45 minutes to further assist the lift-off process. Afterward, the wafer is dipped in acetone and IPA inside the water bath with high power sonication for 10 minutes each sequentially, followed by drying using a nitrogen gun.

Dicing: To protect the wafer from possible contamination in dicing, the wafer is coated with 1.5 μm photo-resist (AZ ECI 3007), where a monolayer of HMDS vapor is deposited before the photo-resist coating, which might remain on the Nb and Si surfaces after the final cleaning, that would prevent re-oxidation of the Nb and Si surface [3]. It is then diced into chips (Disco DAD321) with specific care about the electrostatic discharge potential damages during the dicing.

Final cleaning: After dicing, chips are dipped in remover 1165 inside a 60°C water bath for 30 minutes without sonication. Then, chips are dipped in acetone and IPA solutions inside the water bath for 10 minutes each without sonication, sequentially. Finally, chips are dried with a nitrogen gun.

Packaging: We fix a chip on a sample table using diluted GE varnish with acetone. The chip is then wire-bonded to a PCB with 20-μm diameter Al wires (F&S Bondtec 56i) and covered with an Al lid.

B. Sample investigation

The scanning electron microscope (SEM) images of the Josephson junction in the final devices are shown in the main text. We further investigate the surface properties and topography of the junction, as well as those for the Al-Al and Al-Nb connections using atomic force microscopy (AFM) (see Fig. S1). We measure an average

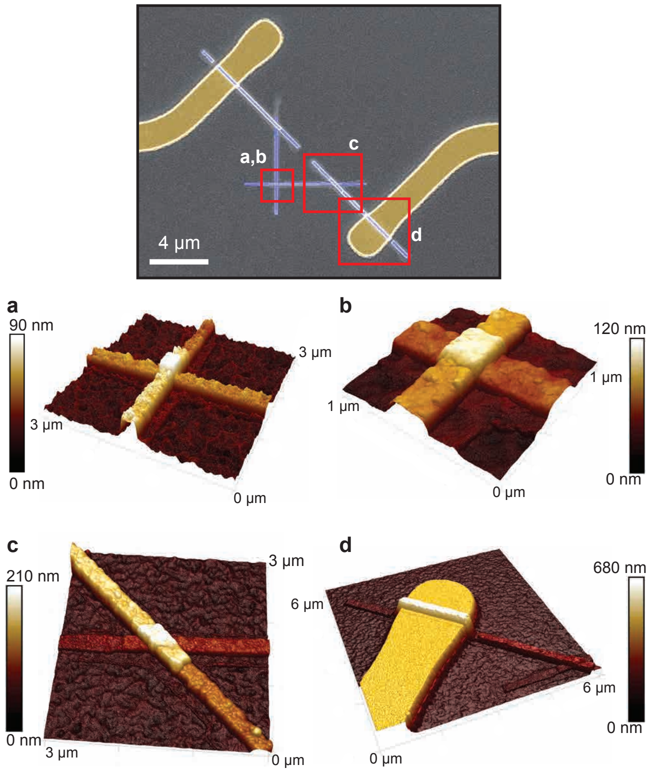

FIG. S1. **Surface investigation on the device.** Atomic force microscopy (AFM) characterizes the surfaces of different parts of the device. (**a**),(**b**) AFM images of the Josephson junction. (**c**),(**d**) AFM images of the Al-Al and Al-Nb connections, respectively.

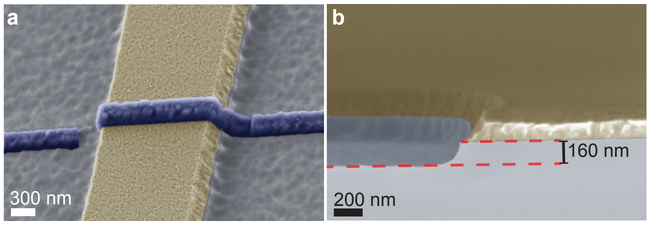

FIG. S2. **Cross section of the etched Nb film on the Si substrate.** (**a**) Tilted SEM image of the Nb-Al connection. (**b**) Cross-sectional SEM image of the Nb-Si interface. Plasma etching of the Nb layer is controlled with time and results in over-etching of the silicon substrate by ≈ 160 nm.

roughness for the silicon surface of $R_{\mathrm{a}}^{(\mathrm{Si})} = 2.6$ nm, which we attribute to the DRIE over-etching of the silicon in the Nb etching process. The roughness of the Nb layer is measured as $R_{\mathrm{a}}^{(\mathrm{Nb})} = 1.1$ nm (Fig. S1d). The step size from the Si surface to the Nb top surface is found to be ~ 325 nm, which includes the Nb layer thickness and the Si over-etched thickness. Figure S2 shows a tilted SEM image of the Nb-Al connection as well as a cross-sectional SEM of the Nb-Si interface. Note that the thickness of the Nb layer in the cross-sectional SEM becomes thinner by the cleaving process than the actual one. Nevertheless, we estimate the depth of the over-etched silicon to be ≈ 160 nm from the cross-sectional SEM. Subtracting this depth from the total step size measured with the AFM, we estimate the actual thickness of the Nb layer to be ≈ 165 nm. Furthermore, the cross-sectional SEM shows that the Nb etching process does not result in silicon undercuts, but realizes a tapered profile, which is useful for making the Nb-Al connection easier and improving the relaxation times [3].

In Figs. S1a and b, there are unknown thin layers visible on the silicon surface around the Al structures. Since the thin layers remain within the undercut of the e-beam resist, this may be due to the re-deposition of the e-beam resist during the argon milling in the shadow evaporation step.

SUPPLEMENTARY NOTE 2. ALLAN DEVIATION ANALYSIS

Allan deviation analysis is an useful technique to identify the source of random fluctuations. In this section, we will first show that two kind of random process mediated by TLS will have identical signature on Allan deviation (ADEV). Next, the ADEV of the long-term qubit parameter fluctuations data are calculated and are fitted by the analytical model.

The first noise process we study is called random telegraph noise, which means a random variable x jumps between two possible values (0 and 1 for simplicity). The dynamics is governed by a set of rate equation,

$$\begin{aligned} P_1(t+dt) &= P_0(t)\gamma_0 dt + P_1(t)\,(1-\gamma_1 dt)\,, \\ P_0(t+dt) &= P_1(t)\gamma_1 dt + P_0(t)\,(1-\gamma_0 dt)\,, \end{aligned} \tag{1}$$

where γ_0 and γ_1 are the transition rates, while $P_0(t)$ and $P_1(t)$ are the probability for random binary variable x to be 0 and 1 in time t, respectively. The process can be understood as a Markovian process between the two values, where the average dwell time in value 0 is $\frac{\gamma_1}{\gamma_0+\gamma_1}$ of the total time, and $\frac{\gamma_0}{\gamma_0+\gamma_1}$ for value 1. In the interacting defect model, the state transitions of a low-frequency TLS with excitation rate γ_0 and relaxation rate γ_1 can change the frequency of a high-frequency TLS coupled to a qubit, resulting in the fluctuation in the qubit relaxation time by random telegraph noise [4].

The second noise process we will consider is a Poisson reset process triggered by events with rate γ. After each event, a random continuous variable x is drawn from a probability distribution, $P(x)$. The random fluctuations caused by gamma and cosmic ray [5–7] can be modeled with this process, where the absorption of ionizing radiation may cause charge rearrangement in a substrate, resulting in the random reset of the frequency of a TLS that induces a qubit decay.

To begin with, we will calculate the auto-correlation for these two process. For the random telegraph noise, where $x = 0$ or 1, the only non-zero contribution in $c_{xx}(\tau)$

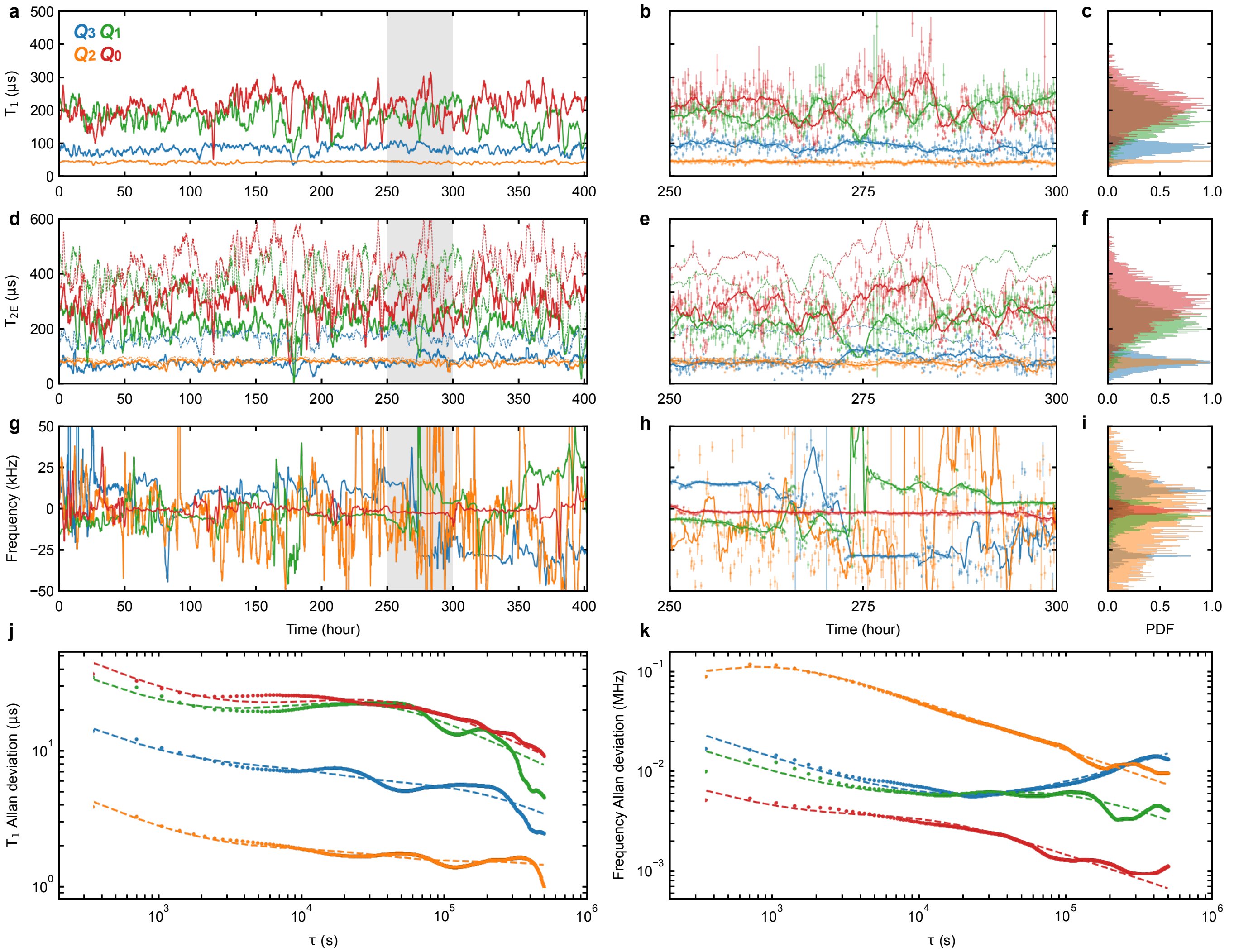

FIG. S3. **Long-term stability of qubit parameters and Allan deviation analysis.** (**a**),(**d**) and (**g**) Relaxation time (T_1), Hahn-echo dephasing time ($T_{2\mathrm{e}}$), and qubit frequency of four qubits Q_0–Q_3 as a function of time. (**b**),(**e**) and (**f**) are the magnified plots for the gray regions in the left panels, respectively. The dots in the magnified plots are the results obtained from the individual time traces, while the solid lines in both plots are their smoothed data with a 5-hour time window. (**c**),(**f**) and (**i**) Histograms for the system parameters shown in the left panels, of four qubits. The dashed lines for $T_{2\mathrm{e}}$ shows the T_1 limit, i.e., $2T_1$. (**j**),(**k**) Allan deviation of relaxation time and qubit frequency, respectively. The dots are the data, while the dashed lines are the fitting results based on the TLS model.

appears when $x(t) = x(t+\tau) = 1$. Therefore, the autocorrelation is written as

$$\begin{aligned} c_{xx}(\tau) &= \langle x(t)x(t+\tau)\rangle \\ &= P\left(x(t)=1\right) P\left(x(t+\tau)=1|x(t)=1\right) \\ &= \frac{\gamma_0}{\gamma_1+\gamma_0} P_{11}(\tau), \end{aligned} \tag{2}$$

where $P\left(x(t)=1\right) = \gamma_0/(\gamma_1+\gamma_0)$ is valid due to the detailed balance condition. For simplicity, we denote $P_{11}(\tau) = P\left(x(t+\tau)=1|x(t)=1\right)$ and $P_{01}(\tau) = P\left(x(t+\tau)=0|x(t)=1\right)$. Furthermore, they should satisfy a set of equations:

$$\begin{aligned} P_{11}(\tau) + P_{01}(\tau) = 1, \\ P_{11}(\tau+d\tau) = P_{11}(\tau)(1-\gamma_1 d\tau) + P_{01}(\tau)\gamma_0 d\tau, \end{aligned} \tag{3}$$

where we take the limit of $d\tau \to 0$ so the chance of having more than 1 switching during $d\tau$ vanishes. Eq. (3) is then transformed in to a differential equation of $P_{11}(\tau)$ as

$$\frac{dP_{11}(\tau)}{d\tau} = -P_{11}(\tau)(\gamma_1+\gamma_0) + \gamma_0, \tag{4}$$

which can readily be solved with the initial condition $P_{11}(0) = 1$ as

$$P_{11}(\tau) = \frac{\gamma_0}{\gamma_0+\gamma_1} + \frac{\gamma_1}{\gamma_0+\gamma_1} e^{-(\gamma_0+\gamma_1)|\tau|}. \tag{5}$$

Substituting the solution into Eq. (2), we obtain $c_{xx}(\tau)$ as

$$c_{xx}(\tau) = \frac{\gamma_0\gamma_1}{(\gamma_0+\gamma_1)^2} e^{-(\gamma_0+\gamma_1)|\tau|}, \tag{6}$$

where we omit the constant part.

For the Poisson reset process, $x(t)$ and $x(t+\tau)$ would be the same value if there are no switching event within time τ, and would be drawn from 2 i.i.d distributions if there are at least 1 switching event within τ. Then, the auto-correlation is obtained as

$$\begin{aligned} c_{xx}(\tau) &= \langle x(t)x(t+\tau)\rangle \\ &= \langle x_0x_1\rangle P(\text{switched within } \tau) \\ &\quad + \langle x_0^2\rangle P(\text{not switched within } \tau) \\ &= \langle x_0x_1\rangle(1-e^{-\gamma|\tau|}) + \langle x_0^2\rangle e^{-\gamma|\tau|}, \end{aligned} \tag{7}$$

where x_0 and x_1 are random variables drawn from 2 i.i.d distributions. For simplicity, we consider $P(x)$ to be a normal distribution of zero mean and σ^2 variance. Hence, the auto-correlation of x for Poisson reset process is calculated as

$$c_{xx}(\tau) = \sigma^2 e^{-\gamma|\tau|}. \tag{8}$$

To summarize, the two noise processes we discussed have the same form in the auto-correlation function, i.e., a double sided exponential function. By applying the Wiener–Khinchin theorem, we obtain a Lorentzian-type power spectral density (PSD) of random variable x as $S_{xx}(\omega) = 2A\frac{\alpha}{\alpha^2+\omega^2}$ for both the noise process, with $\{A = \frac{\gamma_0\gamma_1}{(\gamma_0+\gamma_1)^2}, \alpha = \gamma_0+\gamma_1\}$ for the random telegraph noise and $\{A=\sigma^2, \alpha=\gamma\}$ for the Poisson reset process.

To continue, we calculate the ADEV by following the reference [8]. From the definition of ADEV, we write

$$\begin{aligned} \sigma_x^2(\tau) &= \langle[\bar{x}(t) - \bar{x}(t+\tau)]^2\rangle/2 \\ &= \frac{1}{2\tau^2}\langle[q(t+\tau) - q(t) - q(t+2\tau) + q(t+\tau)]^2\rangle \\ &= \frac{1}{2\tau^2}[2c_{qq}(2\tau) - 8c_{qq}(\tau) + 6c_{qq}(0)], \end{aligned} \tag{9}$$

where we assume the process is stationary. Here $q(t)$ is the phase variable defined as $q(t) = \int_0^t x(\tau)d\tau$, in the sense that we treat $x(t)$ as the frequency variable in the conventional setup for ADEV, i.e., $\frac{dq(t)}{dt} = x(t)$ and $\bar{x}(t) = \int_t^{t+\tau} x(t)dt/\tau = [q(t+\tau) - q(t)]/\tau$.

The PSDs of $q(t)$ and $x(t)$ is related as $S_{qq}(\omega) = S_{xx}(\omega)/\omega^2 = 2A\frac{\alpha}{\omega^2(\alpha^2+\omega^2)}$. Then, we perform an inverse Fourier transform for $S_{qq}(\omega)$ to obtain the auto-correlation for q as

$$c_{qq}(\tau) = -A\frac{e^{-\alpha|t|} + \alpha|t|}{\alpha^2}. \tag{10}$$

Substituting $c_{qq}(\tau)$ into Eq. (9), we obtain the ADEV as

$$\sigma_x(\tau) = \frac{\sqrt{A(4e^{-\alpha t} - e^{-2\alpha t} + 2\alpha t - 3)}}{\tau\alpha}. \tag{11}$$

To characterize the long-term stability of the transmon parameters (frequency, T_1, $T_{2\mathrm{e}}$, etc.), we record the fluctuations of the qubit coherences and frequency over 400 hours, which are shown in Fig. S3a-f. The one cycle consisting of the relaxation, Ramsay, and Hahn-echo sequences is repeated with an interval of about 6 minutes, where the four qubits are simultaneously controlled and read out by frequency multiplexing. Note that the qubit frequencies are obtained by the Ramsey measurements.

To confirm whether these fluctuations are mediated by TLSs with the noise processes we discussed in the last section, we fit the ADEV of qubit T_1 and frequency with the following model:

$$\sigma_x(\tau) = \sigma_{x_1}(\tau) + \sigma_{x_2}(\tau) + k\sqrt{\frac{1}{\tau}}, \tag{12}$$

where $\sigma_{x_1}(\tau)$ and $\sigma_{x_2}(\tau)$ are two ADEV with independent parameters (A and α) based on Eq. (11), while the term $\sqrt{1/\tau}$ is a white noise contribution with a coefficient k. The fitting results are shown in Figs. S3g and H in dashed lines. The fitted lines are consistent with the ADEV data we observed, which implies that the long-term fluctuations are likely to be related to the TLS environment. There are two timescales that can be inferred from the fitting of the T_1-ADEV, from the two terms $\sigma_{x_1}(\tau)$ and $\sigma_{x_2}(\tau)$ respectively, one of which is stable and averaged to around 10^5 s. However, as discussed in the last section, we can not distinguish whether the TLSs are fluctuated by a global Poisson process, e.g. gamma and cosmic rays, or they are fluctuated by the low-frequency TLSs they are coupled to.

SUPPLEMENTARY NOTE 3. DETAILS ON SINGLE-SHOT QUBIT READOUT

A. Optimization

Here, we discuss how to optimize the multiplexed single-shot readout of two of the transmon qubits (Q_0 and Q_1) that are used in the main experiment.

Figures S4a show the qubit-state-dependent reflection phase spectra of the readout resonators for qubits Q_0 and Q_1, respectively. After the preparation of the qubits in the G, E, and F states, we send and measure the multiplexed readout pulse, sweeping the readout frequencies. By fitting the phase of the reflection spectra, we obtain the resonator frequency with the qubit in each state, resulting in the qubit-state dependent frequency shifts. The qubit-state dependent resonator frequency shifts for qubits Q_0 and Q_1 are summarized in Table 1.

Figures S4b show the visibilities as a function of the readout frequency, where the visibility is defined as the distance of the complex amplitudes of the readout signals for the different qubit states. Note that the readout amplitude is set to the one showing the linear resonator response. To distinguish between all the three different

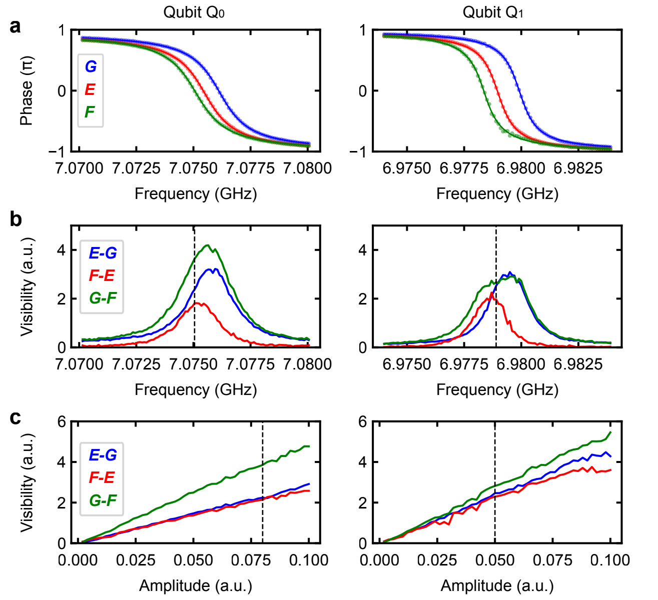

FIG. S4. **Readout optimization for qubits Q_0 (left) and Q_1 (right).** (**a**) Reflection phase spectra of readout resonators with each qubit prepared in the G, E, and F states, respectively. (**b**) Visibilities between E and G, F and E, and G and F as a function of readout frequency. (**c**) Visibilities as a function of readout amplitude. The vertical dashed lines in (**b**) and (**c**) depict the optimized values.

states, the readout frequency is optimized such that the minimal one among the three different visibilities is maximized. The optimized readout frequencies for Q_0 and Q_1 are shown with the dashed lines in Fig. S4b, respectively.

To optimize the readout amplitude, we measure the readout signals for the three different states as a function of the readout amplitude with the optimized readout frequency. Figures S4c show the visibilities as a function of the readout amplitudes for Q_0 and Q_1, respectively. As the readout amplitude increases, the visibilities increase linearly due to the signal-to-noise ratio improvements. However, when the readout amplitude further increases, the visibilities start to show saturations slightly. This is due to the qubit state flip during the readout, caused by the off-resonant drive of the qubit [9]. We choose a readout amplitude that does not show significant saturations but realizes sufficiently large separations between the different states in a single-shot measurement. The optimized readout amplitudes are shown with the dashed lines in Figs. S4c.

B. Characterization

Here, we characterize the single-shot qubit readout for the G, E and F states, which are decomposed into the separation errors and the state-flip errors during the readout while excluding the state-preparation errors.

Since we distinguish between the G, E, and F states using the readout signals in the complex plane, we estimate the separation errors using the 2D histograms of the readout complex amplitudes for the three states. Figures S5a show the scatter plots of the complex amplitudes of the single-shot readout pulses when qubits Q_0 and Q_1 are prepared in the G, E, and F states, respectively. We apply the principal component analysis (PCA) to the raw complex amplitudes in order to maximize the separation between the signals for the G and E states in the real axis (I). This process corresponds to an ordinary linear operation consisting of phase rotation and displacement in the complex plane to maximize the separation in one quadrature axis. In addition, the I and Q quadrature amplitudes are normalized by the I standard deviation for the G state. While both I and Q quadratures are used to distinguish between G, E, and F for the main analysis, for simplicity, only the I quadrature is used to distinguish between G and $\overline{G} = E$ or F for the correlation analysis. Moreover, the I quadrature amplitude for Q_0 can distinguish well between the G, E, and F states, enabling us to characterize the occupation probabilities of the three states using only the histogram of I quadrature amplitude (see Fig. 2f).

To determine the thresholds to distinguish between the G, E, and F states in the complex plane, we fit the distributions of the readout complex amplitudes for the three states to the mixture of three weighted 2D Gaussian functions. This extracts the mean, variance, and covariance of the 2D Gaussian functions for the G, E, and F states, respectively. Using these results, a new sample on the complex plane can be assigned to one of the state clusters that gives the maximum probability of producing this sample, which divides the complex plane into three regions associated with the three states, respectively. The thresholds for qubits Q_0 and Q_1 are shown with the black dashed lines in Figs S5a, respectively.

Using the Gaussian distributions and the thresholds, the separation readout errors ($\varepsilon^{\rm s}_G$ $\varepsilon^{\rm s}_E$, and $\varepsilon^{\rm s}_F$) are defined as the probability of a qubit prepared perfectly in a target state but found in the two other states due to the insufficient signal-to-noise ratio, i.e., the integral of the normalized 2D Gaussian distribution for the target state over the outside of the thresholds. We draw 10^7 samples for each state from the inferred 2D Gaussian distribution, then calculate the probability of being assigned to the non-targeted states to extract the separation error probability numerically. The Monte Carlo integrations lead to the separation errors for the G, E, and F states, i.e., $\varepsilon^{\rm s}_G = 0.061$ % and 0.039 %, $\varepsilon^{\rm s}_E = 0.32$ % and 2.6 %, and $\varepsilon^{\rm s}_F = 0.25$ % and 2.5 % for qubits Q_0 and Q_1, respectively. Note that the finite probabilities experimentally found in the non-targeted state are mainly due to the state-preparation errors, which are dominated by the residual thermal excitation for the G state and the π control errors for the E and F states, respectively. Nevertheless, the state-preparation errors are excluded from the readout errors.

On the other hand, we estimate the state-flip read-

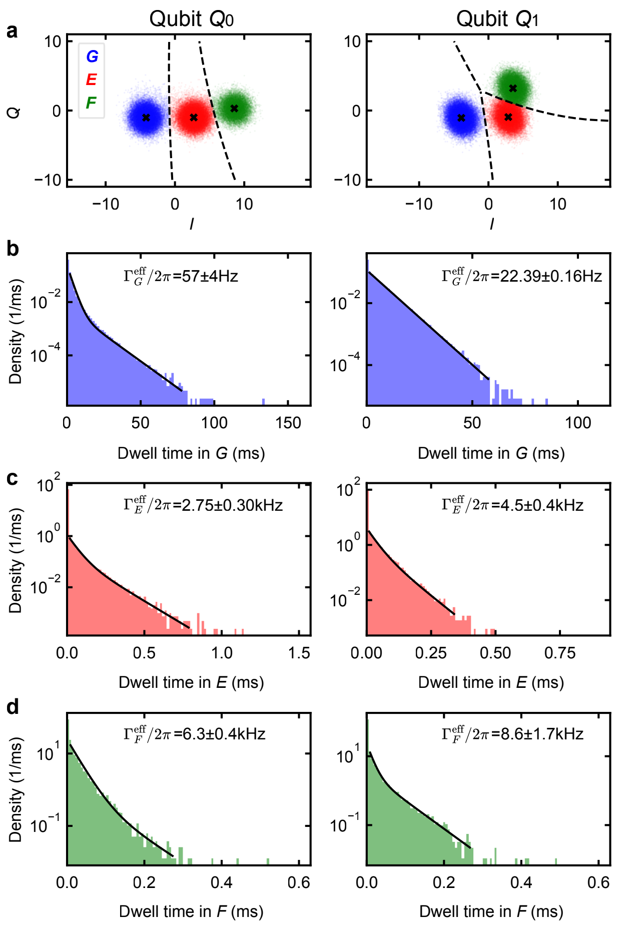

FIG. S5. **Readout characterization for qubits Q_0 (left) and Q_1 (right).** (**a**) Scatter plots of the single-shot readout signals in the complex plane when each qubit is prepared in the G, E, and F states. The dashed lines depict the thresholds to distinguish between the three states. (**b**)–(**d**) Distributions of the dwell times in the G, E and F states, respectively, obtained while the pulse tube effect is minimal. The solid lines are the fitting results, shown within the fitting regions, resulting in the corresponding transition rates. The Q_1 data in panel (**b**) is fitted to a single exponential distribution, while the other data are fitted to mixtures of 2 exponential distributions.

out errors by performing continuous monitoring of the qubit states, with a sequence in which 2.5 μs-long readout pulses are applied successively with an interval of 3 μs (see the pulse scheme in Fig. 4a). As described below in more detail, we use the dwell-time distributions during continuous monitoring to determine the transition rates, resulting in the estimation of the state-flip errors.

However, there are two nontrivial effects of the readout separation errors ($\varepsilon^{\rm s}_{\mathcal{X}}$ for $\mathcal{X} = G, E,$ and F) on the dwell-time distributions and their transition rates. The first effect appears when the initial readout outcome of a dwell event is wrongly assigned, while the second one appears in its complementary case. These two cases are probabilistically mixed in each dwell-time distribution.

For the first case, the separation errors induce fake jump events, where the readout outcomes assign state $\mathcal{Y}$ wrongly as $\mathcal{X}$ due to the separation errors in the first few readout steps successively, and then the following readout assigns the state correctly as $\mathcal{Y}$. In this case, the survival probability that the readout outcomes successively remain in $\mathcal{X}$ until step n is given by $(\varepsilon^{\rm s}_{\mathcal{Y}\to\mathcal{X}})^n$, where $\varepsilon^{\rm s}_{\mathcal{Y}\to\mathcal{X}}$ is the separation error probability that $\mathcal{Y}$ is assigned wrongly as $\mathcal{X}$. This is conveniently rewritten as $\exp(-[-\log(\varepsilon^{\rm s}_{\mathcal{Y}\to\mathcal{X}})/\tau]t)$, where τ is the time interval between two readouts and $t = n\tau$ is the time at step n. Therefore, the characteristic transition rate of such fake initial states is

$$\Gamma^{\rm fake}_{\mathcal{X}} = -\log(\varepsilon^{\rm s}_{\mathcal{Y}\to\mathcal{X}})/\tau. \tag{13}$$

Since the separation error probabilities are sufficiently small, the fake jump events appear as only a sharp peak in the first bin of our dwell-time distribution data (see Figs. S5b–d). Nevertheless, this can be mitigated easily by neglecting the first bin when analyzing the dwell-time distribution.

For the second case, the readout separation errors effectively increase the transition rates since they would change the readout outcomes with an additional probability even though the state remains the same. Here, we consider a trajectory event when the first successive readout steps assign state $\mathcal{X}$ correctly, and then the following readout outcome has been changed to the two other states due to the actual state transition or the separation error. In this case, the survival probability of state $\mathcal{X}$ until step n is given by $(1-\Gamma_{\mathcal{X}}\tau-\varepsilon^{\rm s}_{\mathcal{X}})^n$. The term of $\Gamma_{\mathcal{X}}\tau$ is the probability that the readout outcome is changed by the actual state transition with a rate of $\Gamma_{\mathcal{X}}$ during the readout time τ, while the term of $\varepsilon^{\rm s}_{\mathcal{X}}$ is the probability that the readout outcome is wrongly assigned due to the separation error. Taking the limit of $\tau \to 0$, the survival probability can be approximated as $\exp(-[\Gamma_{\mathcal{X}} + \varepsilon^{\rm s}_{\mathcal{Y}}/\tau]t)$. Therefore, the effective transition rate is found to be

$$\Gamma^{\rm eff}_{\mathcal{X}} = \Gamma_{\mathcal{X}} + \varepsilon^{\rm s}_{\mathcal{Y}}/\tau. \tag{14}$$

This is the transition rate that we can directly extract from each dwell-time distribution through the fitting analysis discussed below. Importantly, since the readout separation errors are independently calibrated by the 2D histograms of the readout complex amplitudes, we can subtract the separation error contributions straightforwardly to extract the actual transition rate as

$$\Gamma_{\mathcal{X}} = \Gamma^{\rm eff}_{\mathcal{X}} - \varepsilon^{\rm s}_{\mathcal{Y}}/\tau. \tag{15}$$

Figures S5b–d show the dwell-time distributions in the G, E, and F states for qubits Q_0 and Q_1, respectively, where we use the time-resolved data during the period with the minimal pulse tube effect (from 0.6 s to 0.7 s in Fig. 4h). Interestingly, we observe dwell-time distributions either with a single characteristic timescale or with two timescales. In the first case, we fit the dwell-time

distribution to an exponential distribution, which has a probability density function (PDF) of $f(\tau) = \Gamma e^{-\Gamma\tau}$. In the second case, we fit the dwell-time distribution to a mixture of two exponential distributions, which has a PDF of $f(\tau) = p\Gamma_1 e^{-\Gamma_1\tau} + (1-p)\Gamma_2 e^{-\Gamma_2\tau}$, and extract the transition rate as a weighted average $\Gamma = p\Gamma_1 + (1-p)\Gamma_2$. For each dwell-time distribution, we apply the two fitting models and select the model that has the lower Bayesian information criterion (BIC). The BIC for model M is defined as $\mathrm{BIC} = k\log(n) - 2\log(\hat{L})$, where k is the number of free parameters of the model, n is the number of data points, and $\hat{L} = p(x|\hat{\theta}, M)$ is the likelihood of producing the observed data x using model M with the optimal parameters $\hat{\theta}$ [10]. As shown in Fig. S5b–d, these fittings extract the effective transition rates containing the effects of the actual transitions and the separation errors, resulting in $\Gamma_G^{\mathrm{eff}}/2\pi = 57 \pm 4$ Hz and 22.39 ± 0.16 Hz, $\Gamma_E^{\mathrm{eff}}/2\pi = 2.75 \pm 0.3$ kHz and 4.5 ± 0.4 kHz, and $\Gamma_F^{\mathrm{eff}}/2\pi = 6.3 \pm 0.4$ kHz and 8.6 ± 1.7 kHz for qubits Q_0 and Q_1, respectively.

As explained above, we can obtain the actual transtion rate as $\Gamma_{\mathcal{X}} = \Gamma_{\mathcal{X}}^{\mathrm{eff}} - \varepsilon_{\mathcal{X}}^{\mathrm{s}}/\tau$ by subtracting the separation error contributions. These result in $\Gamma_G/2\pi = 24.6 \pm 4$ Hz and 1.7 ± 0.16 Hz, $\Gamma_E/2\pi = 2.6 \pm 0.3$ kHz and 3.1 ± 0.4 kHz, $\Gamma_F/2\pi = 6.2 \pm 0.4$ kHz and 7.3 ± 1.7 kHz, for qubits Q_0 and Q_1, respectively. Note that we use the separation errors that are determined using the readout data from continuous monitoring experiments, which yield slightly different values from those for time-separate readout pulses. Using the transition rates during the readout (Γ_G, Γ_E, and Γ_F), we can estimate the upper bound on the state-flip readout error probabilities ($\varepsilon_G^{\mathrm{f}}$ $\varepsilon_E^{\mathrm{f}}$, and $\varepsilon_F^{\mathrm{f}}$). For simplicity, a state-flip readout error can be considered as a transition event occurring within the first half of the total readout time ($\tau = 2.5$ μs). Therefore, the upper bounds can be estimated as $\varepsilon_{\mathcal{X}}^{\mathrm{f}} < \Gamma_{\mathcal{X}}\tau/2$ for $\mathcal{X} = G, E$ and F, leading to $\varepsilon_G^{\mathrm{f}} \lesssim 0.019$ % and 1.3×10^{-3} %, $\varepsilon_E^{\mathrm{f}} \lesssim 2.0$ % and 2.5 %, and $\varepsilon_F^{\mathrm{f}} \lesssim 4.8$ % and 5.7 % for qubits Q_0 and Q_1, respectively.

By using the relaxation times of the E and F states listed in Table 1 and neglecting the thermal contributions, we can roughly estimate the transition rates of the E and F states in the free evolution. This results in $\Gamma_E^{\mathrm{free}}/2\pi \approx 1/T_1/2\pi \approx 0.8$ kHz and $\Gamma_F^{\mathrm{free}}/2\pi \approx 1/T_{1F}/2\pi \approx 1.6$ kHz, respectively. We find that the transition rates during the readout are larger than those in the free evolution, which we attribute to a finite readout backaction, i.e., off-resonant-drive-induced decay [9].

In summary, the readout errors of the G states, primarily determined by separation error, yield $\lesssim 0.08$ % and $\lesssim 0.04$ % for Q_0 and Q_1, respectively. Meanwhile, the errors of the E and F states, primarily influenced by state-flip error, are $\lesssim 2$ % and $\lesssim 5$ % for Q_0, respectively, and $\lesssim 5$ % and $\lesssim 8$ % with additional influence from separation error for Q_1.

SUPPLEMENTARY NOTE 4. DETAILED ANALYSIS FOR TIME-RESOLVED TRANSITION RATES

The transition from one state $\mathcal{X}$ to the other two ($\mathcal{Y}$ and $\mathcal{Z}$) is considered as merging of the two independent Poisson processes with rates of $\Gamma_{\mathcal{X}\to\mathcal{Y}}$ and $\Gamma_{\mathcal{X}\to\mathcal{Z}}$, respectively, where $\mathcal{X}, \mathcal{Y}$, and $\mathcal{Z}$ traverse the three different qubit states (G, E, and F). In a similar manner to the state-flip error characterization, we first obtain the effective transition rates from $\mathcal{X}$ to $\mathcal{Y}$ and $\mathcal{Z}$, and then subtract the readout separation contributions.

The total effective transition rate of the combined process is described by the sum of the two individual processes, i.e., $\Gamma_{\mathcal{X}}^{\mathrm{eff}} = \Gamma_{\mathcal{X}\to\mathcal{Y}}^{\mathrm{eff}} + \Gamma_{\mathcal{X}\to\mathcal{Z}}^{\mathrm{eff}}$. Therefore, the probability of the jump event from $\mathcal{X}$ to $\mathcal{Y}$ can be described as $p_{\mathcal{X}\to\mathcal{Y}} = \Gamma_{\mathcal{X}\to\mathcal{Y}}^{\mathrm{eff}}/\Gamma_{\mathcal{X}}^{\mathrm{eff}}$, and likewise the probability of jumping to $\mathcal{Z}$ can be described as $p_{\mathcal{X}\to\mathcal{Z}} = \Gamma_{\mathcal{X}\to\mathcal{Z}}^{\mathrm{eff}}/\Gamma_{\mathcal{X}}^{\mathrm{eff}}$.

To determine the effective transition rates among the G, E, and F states in a time-resolved fashion, as explained in the main text, we use the dwell-time events that are obtained synchronously with the pulse tube operation and group the dwell events using a bin along the time axis within one pulse tube period, where we adaptively choose the bin width to be proportional to the average G dwell time around the specified time (approximately the minimal time resolution of the transition rate measurement). Next, we obtain the distributions of the dwell time in the G, E, and F states for each time bin, respectively, by accumulating the target dwell events whose event time falls into this time bin. As shown in Figs. S6a–c for Q_0, we infer the effective total rate $\Gamma_{\mathcal{X}}^{\mathrm{eff}}$ by fitting the $\mathcal{X}$ dwell-time distribution to an exponential distribution or a mixture of two exponential distributions, whichever has the lower BIC value, where we do not condition on the final states $\mathcal{Y}$ or $\mathcal{Z}$. We summarize the respective effective transition rates extracted from the time-resolved distributions in Fig. S6d. We show the difference between the BIC values of the two models we used in Fig. S6e, for the three states of Q_0. For the datasets where exponential mixture model are selected, we show the inferred mixture proportion of the fast decay, together with both the fast and the slow transition rates in Fig. S6f and g, respectively.

To infer the $p_{\mathcal{X}\to\mathcal{Y}}$ and $p_{\mathcal{X}\to\mathcal{Z}}$, we count the number of the jump events of $\mathcal{X} \to \mathcal{Y}$ and $\mathcal{X} \to \mathcal{Z}$ out of the total number of the $\mathcal{X}$ dwell events N in each time bin, which lead to the time-resolved binomial probabilities of $p_{\mathcal{X}\to\mathcal{Y}}$ and $p_{\mathcal{X}\to\mathcal{Z}}$, respectively. We show the inferred binomial probabilities in Fig. S6h, where we obtain the statistical error as $\sqrt{Np_{\mathcal{X}\to\mathcal{Y}}(1-p_{\mathcal{X}\to\mathcal{Y}})}$, the standard deviation of the binomial distribution.

By considering the explicit expression of $p_{\mathcal{X}\to\mathcal{Y}} = \Gamma_{\mathcal{X}\to\mathcal{Y}}^{\mathrm{eff}}/\Gamma_{\mathcal{X}}^{\mathrm{eff}}$, we obtain the effective transition rate from $\mathcal{X}$ to $\mathcal{Y}$ by using $\Gamma_{\mathcal{X}\to\mathcal{Y}}^{\mathrm{eff}} = p_{\mathcal{X}\to\mathcal{Y}}\Gamma_{\mathcal{X}}^{\mathrm{eff}}$. Finally, the contribution of the readout separation error is subtracted from the effective transition rate as $\Gamma_{\mathcal{X}\to\mathcal{Y}} = \Gamma_{\mathcal{X}\to\mathcal{Y}}^{\mathrm{eff}} - \varepsilon_{\mathcal{X}\to\mathcal{Y}}^{\mathrm{s}}/\tau$. Similarly, $\Gamma_{\mathcal{X}\to\mathcal{Z}} = \Gamma_{\mathcal{X}\to\mathcal{Z}}^{\mathrm{eff}} - \varepsilon_{\mathcal{X}\to\mathcal{Z}}^{\mathrm{s}}/\tau$. All the results of

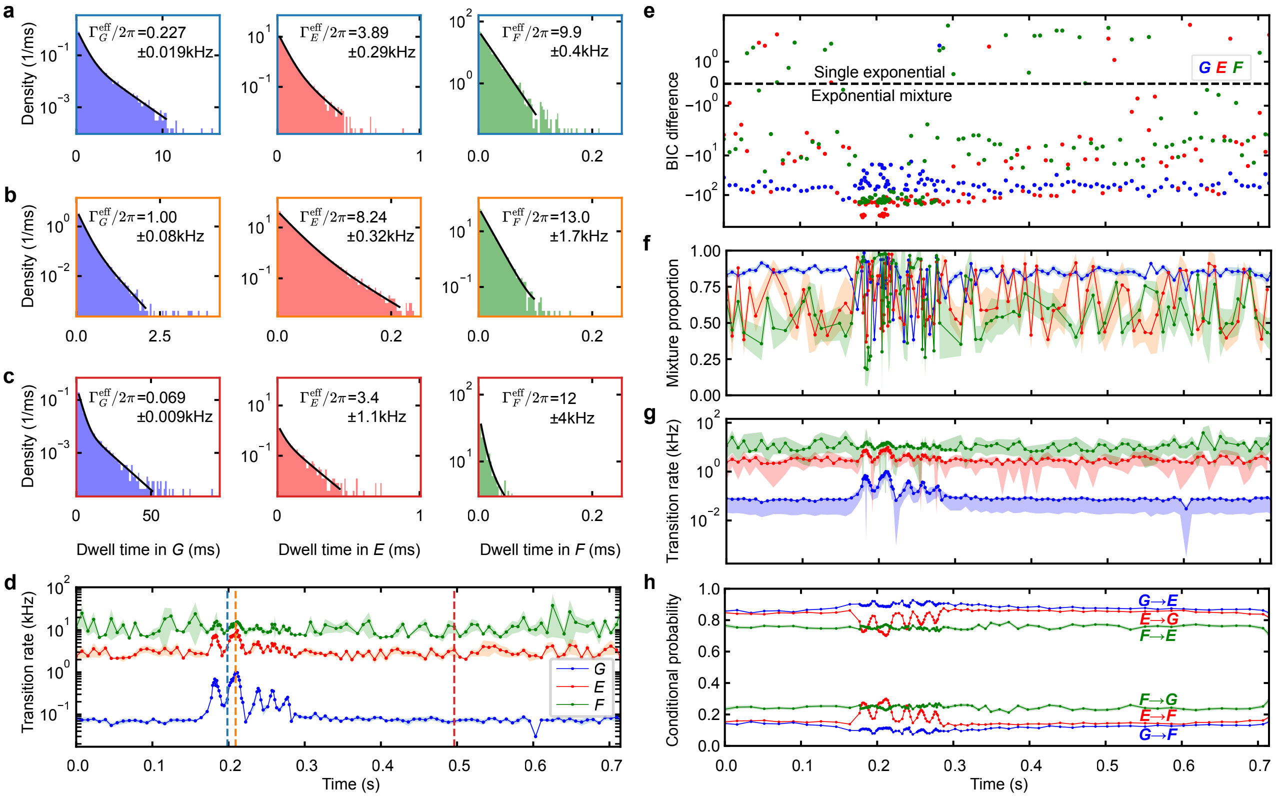

FIG. S6. **Transition rate analysis for qubit Q_0.** (**a**)–(**c**) Time-resolved dwell-time distributions for G, E and F, and for different times specified with dashed lines in (**d**). The solid lines are the fitting results, shown within the fitting regions, resulting in the corresponding transition rates. (**d**) Time-resolved total effective transition rates for the G, E and F states ($\Gamma_G^{\rm eff}$, $\Gamma_E^{\rm eff}$, and $\Gamma_F^{\rm eff}$) as a function of time within one period of the vibrational noise, respectively. (**e**) BIC difference between the model of an exponential distribution and the model of a mixture of two exponential distributions, which is used to select the preferable fitting model for each dwell-time distribution. If the BIC difference is positive, the single exponential model is chosen. (**f**) Time-resolved proportion of the fast exponential decay when the mixture model is adopted based on the BIC. (**g**) Time-resolved transition rates for the three states, inferred from the mixture model. The upper and lower edges of the shaded areas depict the fast and slow transition rates, respectively, while the circles and solid lines in between depict the weighted average values based on the inferred proportion, i.e., the effective transition rates. (**h**) Time-resolved $p_{\mathcal{X}\to\mathcal{Y}}$ inferred from the number of events in each time bin. Note that $p_{\mathcal{X}\to\mathcal{Y}}+p_{\mathcal{X}\to\mathcal{Z}}=1$ when $\mathcal{X},\mathcal{Y}$, and $\mathcal{Z}$ traverse the three different states.

the transition rates among the three states for Q_0 are shown in Fig. 4i of the main text. Note that $\varepsilon^{\rm s}_{\mathcal{X}\to\mathcal{Y}}$ and $\varepsilon^{\rm s}_{\mathcal{X}\to\mathcal{Z}}$ are the separation error probabilities of $\mathcal{X}$ wrongly assigned to $\mathcal{Y}$ and $\mathcal{Z}$ due to the insufficient signal-to-noise ratio, respectively, which are determined based on the Monte Carlo integrations in a similar manner to the characterization of the separation errors. For visualization purpose, we remove the data points from relevant figures, that either the fitting is unsuccessful, or the fitting error is so large that the lower confidence bound is negative.

To further extract information on the qubit thermodynamics, we first derive the steady-state equation, assuming detailed balance condition is satisfied. We denote the steady-state populations of the qubit as $P_\mathcal{X}$ ($\mathcal{X}=G,E$, and F). They should satisfy three homogeneous linear equations

$$0=\frac{{\rm d}P_\mathcal{X}}{{\rm d}t}=-P_\mathcal{X}(\Gamma_{\mathcal{X}\to\mathcal{Y}}+\Gamma_{\mathcal{X}\to\mathcal{Z}})+P_\mathcal{Y}\Gamma_{\mathcal{Y}\to\mathcal{X}}+P_\mathcal{Z}\Gamma_{\mathcal{Z}\to\mathcal{X}}, \tag{16}$$

where $\mathcal{X},\mathcal{Y},\mathcal{Z}$ traverse the three possible states G, E and F. We can rewrite these linear equations in the compact form as $\mathbf{Ax}=\mathbf{b}$, where

$$\mathbf{A}=\begin{pmatrix}\Gamma_{G\to E}+\Gamma_{G\to F} & -\Gamma_{E\to G} & -\Gamma_{F\to G}\\ -\Gamma_{G\to E} & \Gamma_{E\to F}+\Gamma_{E\to G} & -\Gamma_{F\to E}\\ -\Gamma_{G\to F} & -\Gamma_{E\to F} & \Gamma_{F\to G}+\Gamma_{F\to E}\end{pmatrix}, \tag{17}$$

$\mathbf{x}=(P_G,P_E,P_F)^\top$ and $\mathbf{b}=(0,0,0)^\top$. These linear equations automatically have non-zero solutions, since $\det(\mathbf{A})=0$.

Next, we solve the steady-state populations $P_\mathcal{X}$ with the first two rows of Eq. (17) plus the assumption that the

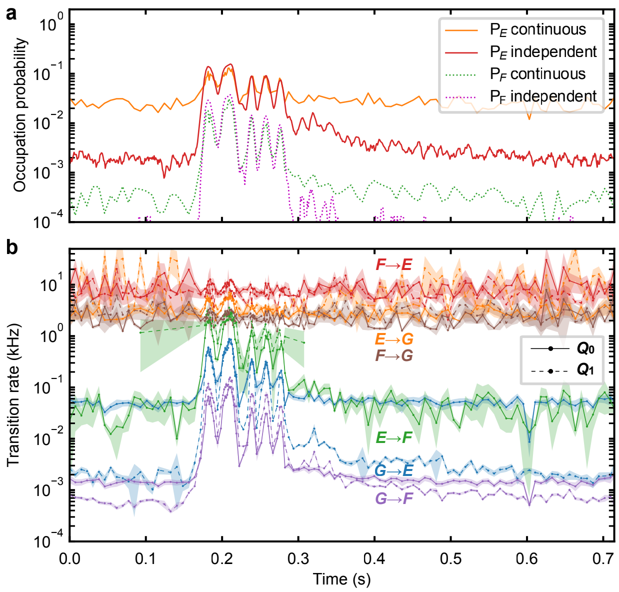

FIG. S7. **Validation of the transition rate inference.** (**a**) Comparison of the state populations obtained by the detailed analysis of the continuous monitoring data, to those obtained by the independent measurement as shown in the Fig. 3d. (**b**) Time-resolved transition rates $\Gamma_{\mathcal{X}\to\mathcal{Y}}$ obtained for both qubits Q_0 and Q_1, where $\mathcal{X}$ and $\mathcal{Y}$ traverse G, E and F. The different initial and final states are labeled with different colors, while the two qubits are labeled with solid or dashed lines, respectively.

qubit is closed within these three states, i.e., $P_G + P_E + P_F = 1$. We show the calculated populations in Fig. S7a, compared to the populations obtained by independent single-shot readout of the qubit, detailed in Fig. 3d. The populations obtained from the detailed balance analysis reproduce those obtained from the independent single-shot readout, while finite discrepancies are observed in the "quiet" period, when the pulse tube effect is minimal, due to the backaction from the continuous monitoring of the qubit [9].

Moreover, we compare the time-resolved transition rates between Q_0 and Q_1 in Fig. S7b. Both qubits show similar nonequilibrium dynamics due to the global nature of the mechanical shock induced by the pulse tube cooler.

As shown in Figs. 4i, we observe substantial direct transition rates between G and F states. To verify whether it is due to the limited time resolution of the measurement, we estimate the effective transition rates induced by a cascaded decay process, i.e., consecutive G-E and E-F transitions in a single readout step. For example, a $G \to E$ transition followed by an $E \to F$ transition within the readout interval of $\tau = 3$ μs can be misrecognized as a direct $G \to F$ transition in our analysis. An upper bound of the transition rate of the time-unresolved cascaded process is $(\Gamma_{G\to E}\tau) \times (\Gamma_{E\to F}\tau)/\tau = \Gamma_{G\to E}\Gamma_{E\to F}\tau$, which is the probability of both transitions happening once within the readout interval τ, divided by τ. We calculate this upper bound for both qubits and for both $G \to F$ and $F \to G$ transitions, confirming that the cascaded transition rates are much smaller than the experimentally observed direct transition rates. This implies that the direct G-F and F-G transitions can actually exist possibly due to readout-induced higher-order transitions [11] or auxiliary mode-assisted two-photon transitions [12].

SUPPLEMENTARY NOTE 5. CALIBRATION OF QUANTUM EFFICIENCY OF MICROWAVE MEASUREMENT

Here, we explain how we calibrate our microwave measurement chain including circulators/isolators, JTWPA, HEMT amplifier, etc. The calibration ensures the measurement efficiency of microwave background noise is nearly quantum-limited, allowing us to conclude that there is no dominant electrical noise that would electrically excite superconducting qubits in our experiment.

First, we calibrate the input attenuation from room temperature to the sample in the dilution refrigerator. To this end, we use the photon-number resolved qubit spectra to determine the readout photon flux at the sample. Figure S8 shows the two-tone spectroscopy for qubits Q_2 and Q_3, i.e., the quadrature of a fixed-frequency readout signal as a function of a qubit drive frequency. The presented quadrature is obtained as the result of the principle component analysis on the readout complex amplitude to maximize the signal-to-noise ratio (SNR). These results show well-resolved photon number splitting, which is sensitive to the readout photon number flux to be calibrated here. It is worth mentioning that qubits Q_0 and Q_1 do not show clear photon number splitting features, which are not useful for this analysis.

We fit the experimental results to the numerical simulation results based on the Lindblad master equation:

$$\dot{\rho} = -i[\hat{H}/\hbar, \rho] + \kappa\mathcal{D}[\hat{a}] + \gamma\mathcal{D}[\hat{\sigma}] + \gamma_\varphi\mathcal{D}[\hat{\sigma}_z/2], \qquad (18)$$

where $\hat{\rho}$ is the density matrix, $\hat{a}$ and $\hat{\sigma}$ are the lowering operators for the readout cavity and qubit, respectively, $\hat{\sigma}_z$ is the Pauli z operator, $\kappa = \kappa_{\rm ex} + \kappa_{\rm in}$ is the cavity total decay rate, $\gamma = 1/T_1$ is the qubit relaxation rate, and $\gamma_\varphi = 1/T_{2*} - 1/(2T_1)$ is the qubit pure dephasing rate. The Hamiltonian in the rotating frames for the cavity and the qubit at the readout and drive frequencies ($\omega_{\rm r}$ and $\omega_{\rm d}$), respectively, is described as

$$\begin{aligned}\hat{H}/\hbar =& (\omega_{\rm c} - \omega_{\rm r})\hat{a}^\dagger\hat{a} + \frac{\omega_{\rm q} - \omega_{\rm d}}{2}\hat{\sigma}_z - \chi\hat{a}^\dagger\hat{a}\hat{\sigma}_z \\ &+ \frac{\Omega_{\rm r}}{2}(\hat{a} + \hat{a}^\dagger) + \frac{\Omega_{\rm d}}{2}(\hat{\sigma} + \hat{\sigma}^\dagger),\end{aligned} \qquad (19)$$

where $\omega_{\rm c}$ and $\omega_{\rm q}$ are the cavity and qubit frequencies, $\chi = \chi_{GE}/2$ is the state-dependent frequency shift, $\Omega_{\rm r}$ and $\Omega_{\rm d}$ are the readout and drive amplitudes, respectively. We calculate the steady-state solution of the cavity complex amplitude $\langle\hat{a}\rangle = {\rm Tr}[\hat{a}\hat{\rho}]$ as a function of $\omega_{\rm d}$.

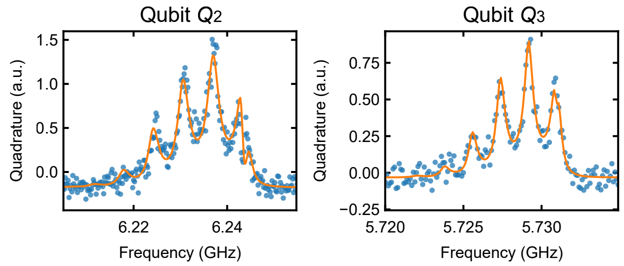

FIG. S8. **Photon-number resolved qubit spectra for qubits Q_2 (left) and Q_3 (right).** The blue points and the orange solid lines are the experimental results and the fitting results based on the numerical simulations, respectively.

According to the experiment, we set $\omega_r = \omega_c + \chi$ and apply the principle component analysis to the numerically obtained cavity field to obtain the quadrature. As shown with the orange solid lines in Fig. S8, we fit the experimental results to the numerical simulation results with fitting parameters of ω_q, χ, Ω_r, Ω_d, and the scaling and offset factors, while the other parameters are fixed at the values listed in Table 1. From the fitting results, we estimate the readout photon flux at the cavity, $\dot{n}_r$, by using the relation of $\Omega_r = 2\sqrt{\kappa_{ex}\dot{n}_r}$. By comparing this with the readout photon flux at room temperature, we obtain the total attenuation between the sample and the input port of the dilution refrigerator at the readout frequencies for qubits Q_2 and Q_3 as $R = -82.6$ dB and -85.7 dB, respectively. The difference would be explained by the impedance mismatch in the input line, since it is comparable with the transmission ripples within the range of the readout frequencies. Therefore, we determine the attenuation ratio to be $R = -84 \pm 2$ dB. Note that we used the nominal attenuation of the attenuators and coaxial cables at room temperature and the nominal output power of the vector network analyzer to estimate the photon flux at the input port of the refrigerator.

Next, we estimate the readout-induced dephasing rates of qubits Q_0 and Q_1, which are mainly used for the time-domain experiments in this paper. The dephasing rate is theoretically described as

$$\gamma_\varphi^r = \frac{\kappa\chi^2}{(\omega_c-\omega_r)^2+\chi^2+(\kappa/2)^2}\,(n_r^G+n_r^E), \tag{20}$$

where $n_r^{G/E} = \kappa_{ex}\dot{n}_r/((\omega_c \pm \chi - \omega_r)^2 + (\kappa/2)^2)$ is the average photon number in the readout cavity with the qubit in the G/E state [13]. By combining the calibrated attenuation ratio in the refrigerator with the readout photon flux at the input port of the refrigerator, we estimate the readout photon flux at the sample, resulting in the readout-induced dephasing rates for qubits Q_0 and Q_1, $\gamma_\varphi^r/2\pi = 1.2 \pm 0.4$ MHz and 1.0 ± 0.3 MHz, respectively.

Following Ref. [13], we associate the readout-induced dephasing rate with the quantum efficiency of the microwave measurement, denoted by η. Here, we can simply consider the full microwave measurement chain (see Fig. 8) as a single quadrature amplitude measurement with a quantum efficiency of η, or a perfect single quadrature measurement after a loss of η. In contrast to the explicit expression of Eq. (20), the readout-induced dephasing rate can be also described as

$$\gamma_\varphi^r = \frac{\kappa}{2}\left|\alpha_c^G - \alpha_c^E\right|^2, \tag{21}$$

where $\alpha_c^{G/E}$ is the steady-state coherent readout amplitude of the cavity with the qubit in the G/E state. Using the input-output relation, $\hat{a}_{out} = \hat{a}_{in} - \sqrt{\kappa_{ex}}\hat{a}$, the dephasing rate is rewritten as

$$\gamma_\varphi^r = \frac{\kappa}{2\kappa_{ex}}\left|\alpha_{out}^G - \alpha_{out}^E\right|^2, \tag{22}$$

where $\hat{a}_{in}$ and $\hat{a}_{out}$ are the input and output quantum fields for the readout cavity, respectively, and $\alpha_{out}^{G/E}$ is the coherent amplitude of the cavity output field, i.e. $\langle\hat{a}_{out}\rangle$, when the qubit is in the G/E state. To effectively take account into the quantum efficiency of the measurement, the cavity output field is mixed with an ancilla mode ($\hat{a}_{ancilla}$) in the vacuum mode as $\hat{a}'_{out} = \sqrt{\eta}\hat{a}_{out} + \sqrt{1-\eta}\hat{a}_{ancilla}$. Thus, the dephasing rate can be further modified as

$$\gamma_\varphi^r = \frac{\kappa}{2\kappa_{ex}\eta}\left|\alpha'^G_{out} - \alpha'^E_{out}\right|^2, \tag{23}$$

where $\alpha'^{G/E}_{out} = \sqrt{\eta}\alpha^{G/E}_{out}$ is the coherent amplitude of the cavity output field after the loss, i.e. $\langle\hat{a}'_{out}\rangle$, when the qubit is in the G/E state.

Here, the expression on the right-hand side of Eq. (23) can be described by the signal-to-noise ratio (SNR) rate of the qubit readout quadrature outcomes. The cavity output field, $\hat{a}'_{out}$, is demodulated by a normalized square-shaped window function with a demodulation length of τ:

$$w(t) = \begin{cases} \frac{1}{\sqrt{\tau}}, & \text{if } 0 \le t \le \tau \\ 0, & \text{otherwise.} \end{cases} \tag{24}$$

Note that the cavity output field and the demodulation window function are described in the rotating frame of the readout frequency. Using this window function, the demodulated mode is defined as

$$\hat{A} = \int dt\, w(t)\hat{a}'_{out}, \tag{25}$$

which satisfies $[\hat{A}, \hat{A}^\dagger] = 1$. Then, the readout quadrature is obtained as

$$\hat{I} = \frac{\hat{A}e^{i\theta} + \hat{A}^\dagger e^{-i\theta}}{\sqrt{2}}, \tag{26}$$

where the quadrature projection phase θ is optimized to maximize the SNR. Here, the SNR of the qubit readout is defined as

$$\mathrm{SNR} = \frac{\left(\langle\hat{I}\rangle_G - \langle\hat{I}\rangle_E\right)^2}{\langle\hat{N}\rangle_G + \langle\hat{N}\rangle_E}, \tag{27}$$

where $\langle \hat{I} \rangle_{G/E}$ and $\langle \hat{N} \rangle_{G/E} = \left(\langle \hat{I}^2 \rangle_{G/E} - \langle \hat{I} \rangle^2_{G/E} \right)$ are the expectation and variance of the readout quadrature when the qubit in the G/E state, respectively.

Using $\max \left[\left| \langle \hat{I} \rangle_G - \langle \hat{I} \rangle_E \right| \right] = \sqrt{2\tau} |\alpha'^G_{\text{out}} - \alpha'^E_{\text{out}}|$ and $\langle \hat{N} \rangle_G = \langle \hat{N} \rangle_E = 1/2$, the SNR is simplified as

$$\text{SNR} = 2\tau |\alpha'^G_{\text{out}} - \alpha'^E_{\text{out}}|^2. \tag{28}$$

This gives the SNR rate as

$$\gamma_{\text{SNR}} = \frac{\text{SNR}}{\tau} = 2|\alpha'^G_{\text{out}} - \alpha'^E_{\text{out}}|^2. \tag{29}$$

By comparing this with Eq. (23), we obtain

$$\eta = \frac{\kappa}{\kappa_{\text{ex}}} \frac{\gamma_{\text{SNR}}}{4\gamma^{\text{r}}_{\varphi}}. \tag{30}$$

Note that our expression has the correction of the cavity collection efficiency when compared to the ones found in Refs. [13, 14].

By following the definition in Eq. (27) and using the I quadrature data for the G and E states shown in Fig. S5, we experimentally obtain the SNR of the single-shot readout for qubits Q_0 and Q_1 to be SNR = 23.0 and 23.4, respectively. Given the demodulation length of 2.3 μs, the SNR rates are found to be $\gamma_{\text{SNR}}/2\pi = (\text{SNR})/\tau/2\pi = 1.59$ MHz and 1.62 MHz, respectively. Note that the readout and demodulation lengths are sufficiently longer than the cavity time scale ($\approx 1/\kappa$), allowing us to assume that the cavity is in a steady state during the readout, validating our analysis.

Using Eq. (30), we estimate the quantum efficiency of our measurement chain at the readout frequencies for qubits Q_0 and Q_1 as $\eta = 0.34 \pm 0.1$ and 0.45 ± 0.2, respectively. Note that the JTWPA is working as a nearly quantum-limited phase-sensitive amplifier, limiting the quantum efficiency of ≤ 0.5. From these results, we can assume that the quantum efficiency of the microwave background noise measurements, shown in Fig. 6, is at least $\eta > 0.2$.

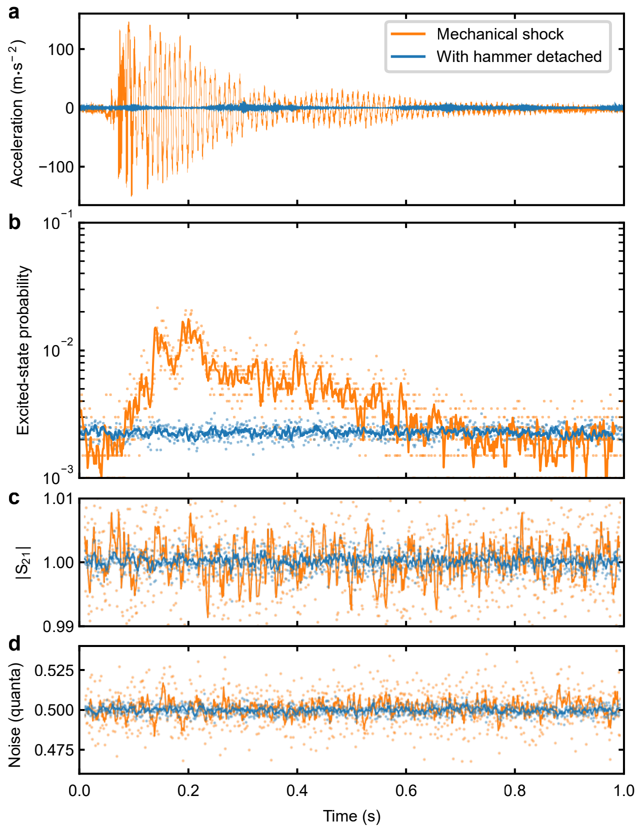

FIG. S9. **Effect of electric noise generated by electric hammer.** (**a**) Time traces of the acceleration signals with the electric hammer activated (orange) and detached (blue), respectively. (**b**) Time-resolved qubit excited-state probabilities, synchronized with the trigger signal to activate the electric hammer. (**c,d**) Time-resolved transmission coefficient and background noise of the full measurement chain, measured synchronously with the referential periodic vibrational noise (orange). The transmission and background noise are normalized by the non-time-resolved values, respectively. Moreover, the background noise is rescaled to the vacuum noise level (1/2), to be in the photon unit. The dots and lines are the raw data and their smoothed data with a 5-ms time window, respectively.

SUPPLEMENTARY NOTE 6. EFFECT OF ELECTRICAL NOISE FROM ELECTRIC HAMMER

In the main text, we show that superconducting qubits are excited by controlled mechanical shocks generated by an electric hammer. However, it should be confirmed that such excitation is purely mechanical and not electrical.

Here, we redo the time-resolved qubit excited-state probability measurement that is synchronized with mechanical shocks controlled by the electric hammer, where we focus on qubit Q_0 of the same device used in the main text but in a different cooling down. For simplicity, we do not distinguish between the E and F states in this measurement. In contrast to the results of the main text, the pulse tube cooler is kept working during the measurement for better stability. Importantly, since the qubit sequences are not synchronized with the pulse tube operation in this specific experiment, possible effects of the pulse tube on the qubit are averaged out, adding a small offset to the excited-state probability in the time-resolved analysis. As shown with the orange data in Figs. S9a and b, the time-resolved qubit excited-state probability reproduces the data shown in Fig. 6 of the main text.

In addition, we apply coherent pulses at around 7 GHz and measure the transmission coefficient and the background noise of the microwave measurement chain in a time-resolved fashion, synchronously with the controlled mechanical shock. In a similar manner to the measure-

ment for Fig. 7 of the main text, this allows us to characterize the effect of the mechanical shocks on the qubit microwave environment. As shown with the orange data in Figs. S9c and d, there is no time variation in the time-resolved data of the transmission coefficient and the background noise. This can exclude explanations that superconducting qubits are excited by possible electrical noise generated by the electric hammer and mechanically induced local heating and triboelectric effect in the qubit environment.

To further convincingly exclude possible effect of electrical noise generated by the electric hammer, we repeat the same experiment, except for the hammer detached from the top of the refrigerator. In this case, a trigger signal from the qubit measurement setup is still sent to the electric hammer to activate it, but it does not generate a mechanical shock. The blue data in Fig. S9 show the results with the hammer detached, confirming the electric hammer operation does not generate any electrical noise that can affect the qubits and the microwave bath. Note that a small vibration noise generated by the pulse tube is observed in Fig. S9a when the electric hammer is detached, which is not synchronized with the present qubit measurement, confirming that its effect cannot influence the time-resolved results.

These results further support our discussions about the mechanical sensitivity of superconducting qubits in the main text.

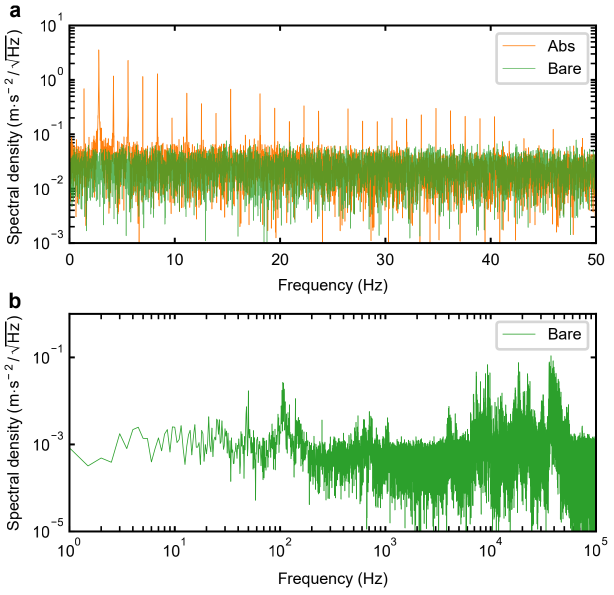

FIG. S10. **Spectral density of vibrational noise generated by the pulse tube cooler in low (a) and high (b) frequency ranges.** The green and orange solid lines are the spectral densities of the bare acceleration data and that of its absolute value, respectively.

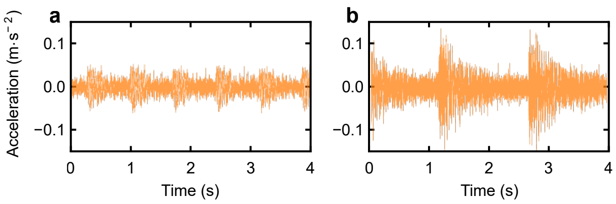

FIG. S11. **Time traces of the acceleration of vibration noise at the qubit stage at room temperature.** (**a**) Vibration noise generated by the pulse tube cooler. (**b**) Vibration noise generated by the electric hammer.

SUPPLEMENTARY NOTE 7. DETAILED ANALYSIS OF VIBRATIONAL NOISE

Here, we show a more detailed analysis of the vibrational noise generated by the pulse tube cooler and the electric hammer.

Figure S10 shows the amplitude spectral densities of the vibrational noise generated by the pulse tube, where the spectra in (a) and (b) use 100-s and 2-s long vibrational time traces at sampling rates of 10 kHz and 500 kHz, respectively. The green data is the amplitude spectral of the raw acceleration time trace, while the orange data is the spectrum of the absolute value of the time trace. The phases of periodic mechanical shocks generated by the pulse tube are random, refraining us from obtaining the fundamental frequency of the pulse tube operation using the bare accelerating data (see the green data in Fig. S10a). Instead, the periodicity of the pulse tube operation can be extracted by taking the absolute value of the bare data, followed by the spectral analysis (see the orange data). This analysis is used for generating the spectral density of the pulse tube vibrational noise shown in Fig. 2 of the main text to compare it with the periodicity of the qubit excitations.

Figure S10b shows the amplitude spectral density of the bare vibrational noise up to 100 kHz, where it shows finite noise components in the high-frequency range. Although the accelerometer works up to 15 kHz from the specification, it is able to convert acceleration to voltage up to 50 kHz to a certain extent. However, this would be still challenging to see which frequency component can be responsible for the nonequilibrium qubit dynamics from the presented data. We believe that giga-hertz or higher frequency vibrational noise, which cannot be characterized by the accelerometer, is more relevant for the mechanically induced qubit excitation.

The acceleration data shown throughout the paper are measured by the accelerometer attached to the top of the dilution refrigerator. However, what matters is the acceleration of the vibrational noise at the qubit stage. While it would be challenging to measure the on-chip vibration noise using the commercially available accelerometer during the operation of the dilution refrigerator, we can easily measure it at room temperature when the refrigerator is warmed up and open. Figures S11a and b show the

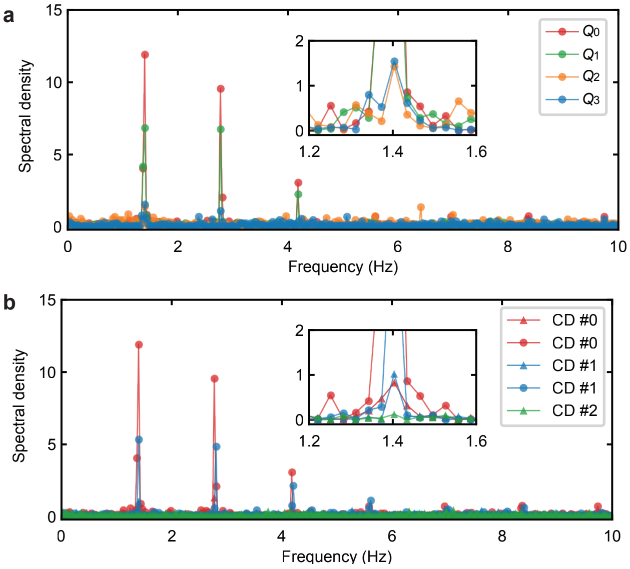

FIG. S12. **Mechanical sensitivities for different qubits and for different cooling downs.** (**a**) Amplitude spectral densities (ASD) of the single-shot quadrature time traces for four qubits Q_0–Q_3. (**b**) ASDs of the single-shot quadrature time traces for Q_0, measured in different cooling downs (CDs #0–2) with the same cryogenic wiring for the DUT. The data in the same color, but with different symbols, show the ones measured in the same cooling down, but at different timing. To be fair, the separations between the readout signals for the G and E states are always normalized to be 1 before obtaining the ASDs in this analysis.

time traces of the acceleration of the one-chip vibrational noises generated by the pulse tube cooler and the electric hammer, respectively. As shown in Fig. S11a, only one of the two operation phases of the pulse tube shows a significant peak in the time trace of the vibrational noise, providing the indirect reason why only the gas-flow phase can contribute to the qubit nonequilibrium dynamics. This is in contrast to the acceleration data that are taken at the top of the refrigerator and show similar vibrational noise in both the gas-flow-in and out phases (see Fig. 3b). Furthermore, the strength of the pulse tube vibrational noise is comparable to or slightly less than that of the electric hammer (Fig. S11b), which is not consistent with the significantly larger qubit excitation induced by the pulse tube cooler than the electric hammer. We think that the qubits are excited by gigahertz or higher frequency vibrational noise, which cannot be easily characterized by accelerometers.

SUPPLEMENTARY NOTE 8. MECHANICAL SENSITIVITIES FOR DIFFERENT QUBITS AND FOR DIFFERENT COOLING DOWNS

Here, we study the dependence of the mechanical sensitivity on different qubits and different cooling downs (CDs). To see the mechanical sensitivity of the qubits, we show the amplitude spectral density (ASD) of the time trance of single-shot qubit readout outcomes while the pulse tube cooler is activated, as shown in Fig. 2e. If the ASD shows larger harmonics with a fundamental frequency of about 1.4 Hz, the qubit is more sensitive to the mechanical shock generated by the pulse tube cooler.

Figure S12a shows the ASDs of the single-shot readout time traces for the four different qubits. It clearly shows that the longer-lived qubits (Q_0 and Q_1) are significantly more sensitive to the mechanical shock than the shorter-lived qubits (Q_2 and Q_3). This implies that the relaxation times of the longer-lived qubits are limited by mechanical sensitive baths, while those of the shorter-lived qubits are limited by other loss mechanisms.

Figure S12b shows the ASDs of the single-shot readout time trace of qubit Q_0 for three different cooling downs (CDs) of the dilution refrigerator. Importantly, the cryogenic wiring for the four-qubit device is kept exactly the same, while other cryogenic wirings that are completely isolated from the main experiment are changed every cooling down. Nevertheless, the mechanical sensitivity of the qubit is varying depending on cooling downs. Interestingly, the sensitivity changes even within the same cooling down (see the results in CDs #0 and #1). This is consistent with the qubit parameter fluctuations due to the TLS baths (see Figs S3). In particular, the ASD for CD #2 shows no mechanical sensitivity, although we don't see any crucial changes in the cryogenic wiring.

One unique setup for our dilution refrigerator is a fiber base optical setup at the still stage, where we are testing electro-optical converter chips via optical fiber coupling with epoxy glue. However, even though the optical setup is kept exactly the same between CDs #1 and #2, the mechanical sensitivity are completely different between these two cooling downs. This may imply that the optical setup does not play an important role in the mechanical sensitivity of the qubit. This is also consistent with that we observe a finite mechanical sensitivity of the qubits even during a cooling down in the absence of samples connected to the optical fibers.

[1] J. M. Kreikebaum, *Superconducting Qubit Enabled Single Microwave Photon Detection* (University of California, Berkeley, 2020).

[2] A. Osman, J. Simon, A. Bengtsson, S. Kosen, P. Krantz, D. P. Lozano, M. Scigliuzzo, P. Delsing, J. Bylander, and A. Fadavi Roudsari, Simplified Josephson-junction fabrication process for reproducibly high-performance superconducting qubits, Applied Physics Letters **118**, 064002 (2021).

[3] A. Nersisyan, S. Poletto, N. Alidoust, R. Manenti,

R. Renzas, C.-V. Bui, K. Vu, T. Whyland, Y. Mohan, E. A. Sete, *et al.*, Manufacturing low dissipation superconducting quantum processors, in *2019 IEEE international electron devices meeting (IEDM)* (IEEE, 2019) pp. 31–1.

[4] C. Müller, J. H. Cole, and J. Lisenfeld, Towards understanding two-level-systems in amorphous solids: insights from quantum circuits, Reports on Progress in Physics **82**, 124501 (2019).

[5] M. McEwen, L. Faoro, K. Arya, A. Dunsworth, T. Huang, S. Kim, B. Burkett, A. Fowler, F. Arute, J. C. Bardin, *et al.*, Resolving catastrophic error bursts from cosmic rays in large arrays of superconducting qubits, Nature Physics **18**, 107 (2022).

[6] T. Thorbeck, A. Eddins, I. Lauer, D. T. McClure, and M. Carroll, TLS Dynamics in a Superconducting Qubit Due to Background Ionizing Radiation, arXiv preprint arXiv:2210.04780 (2022).

[7] C. D. Wilen, S. Abdullah, N. Kurinsky, C. Stanford, L. Cardani, G. d'Imperio, C. Tomei, L. Faoro, L. Ioffe, C. Liu, *et al.*, Correlated charge noise and relaxation errors in superconducting qubits, Nature **594**, 369 (2021).

[8] C. M. Van Vliet and P. H. Handel, A new transform theorem for stochastic processes with special application to counting statistics, Physica A: Statistical Mechanics and its Applications **113**, 261 (1982).

[9] M. Boissonneault, J. M. Gambetta, and A. Blais, Dispersive regime of circuit QED: Photon-dependent qubit dephasing and relaxation rates, Physical Review A **79**, 013819 (2009).

[10] G. Schwarz, Estimating the dimension of a model, The Annals of Statistics **6**, 461 (1978).

[11] D. Sank, Z. Chen, M. Khezri, J. Kelly, R. Barends, B. Campbell, Y. Chen, B. Chiaro, A. Dunsworth, A. Fowler, *et al.*, Measurement-induced state transitions in a superconducting qubit: Beyond the rotating wave approximation, Physical review letters **117**, 190503 (2016).

[12] M. J. Peterer, S. J. Bader, X. Jin, F. Yan, A. Kamal, T. J. Gudmundsen, P. J. Leek, T. P. Orlando, W. D. Oliver, and S. Gustavsson, Coherence and decay of higher energy levels of a superconducting transmon qubit, Physical review letters **114**, 010501 (2015).

[13] J. Gambetta, A. Blais, D. I. Schuster, A. Wallraff, L. Frunzio, J. Majer, M. H. Devoret, S. M. Girvin, and R. J. Schoelkopf, Qubit-photon interactions in a cavity: Measurement-induced dephasing and number splitting, Physical Review A **74**, 042318 (2006).

[14] C. C. Bultink, B. Tarasinski, N. Haandbæk, S. Poletto, N. Haider, D. Michalak, A. Bruno, and L. DiCarlo, General method for extracting the quantum efficiency of dispersive qubit readout in circuit QED, Applied Physics Letters **112** (2018).
